# In hot and cold water: differential life‐history traits are key to success in contrasting thermal deep‐sea environments

**DOI:** 10.1111/1365-2656.12337

**Published:** 2015-03-02

**Authors:** Leigh Marsh, Jonathan T. Copley, Paul A. Tyler, Sven Thatje

**Affiliations:** ^1^ Ocean and Earth ScienceUniversity of SouthamptonNational Oceanography Centre SouthamptonEuropean WaySouthamptonSO14 3ZHUK

**Keywords:** hydrothermal vent, invertebrate reproduction, life‐history biology, remotely operated vehicle, Southern Ocean, thermal adaptation

## Abstract

1. Few species of reptant decapod crustaceans thrive in the cold‐stenothermal waters of the Southern Ocean. However, abundant populations of a new species of anomuran crab, *Kiwa tyleri*, occur at hydrothermal vent fields on the East Scotia Ridge.
2. As a result of local thermal conditions at the vents, these crabs are not restricted by the physiological limits that otherwise exclude reptant decapods south of the polar front.
3. We reveal the adult life history of this species by piecing together variation in microdistribution, body size frequency, sex ratio, and ovarian and embryonic development, which indicates a pattern in the distribution of female Kiwaidae in relation to their reproductive development.
4. High‐density ‘*Kiwa*’ assemblages observed in close proximity to sources of vent fluids are constrained by the thermal limit of elevated temperatures and the availability of resources for chemosynthetic nutrition. Although adult Kiwaidae depend on epibiotic chemosynthetic bacteria for nutrition, females move offsite after extrusion of their eggs to protect brooding embryos from the chemically harsh, thermally fluctuating vent environment. Consequently, brooding females in the periphery of the vent field are in turn restricted by low‐temperature physiological boundaries of the deep‐water Southern Ocean environment. Females have a high reproductive investment in few, large, yolky eggs, facilitating full lecithotrophy, with the release of larvae prolonged, and asynchronous. After embryos are released, larvae are reliant on locating isolated active areas of hydrothermal flow in order to settle and survive as chemosynthetic adults. Where the cold water restricts the ability of all adult stages to migrate over long distances, these low temperatures may facilitate the larvae in the location of vent sites by extending the larval development period through hypometabolism.
5. These differential life‐history adaptations to contrasting thermal environments lead to a disjunct life history among males and females of *K. tyleri*, which is key to their success in the Southern Ocean vent environment.
6. We highlight the complexity in understanding the importance of life‐history biology, in combination with environmental, ecological and physiological factors contributing to the overall global distribution of vent‐endemic species.

## Introduction

The abundant assemblages of fauna at deep‐sea hydrothermal vents are largely sustained by microbial primary production based on the oxidation of reduced inorganic compounds in hydrothermal fluids. Globally, the faunal assemblages at hydrothermal vents exhibit significant variation in taxonomic composition, with several biogeographic provinces recognized among species endemic to deep‐sea chemosynthetic environments during the adult phase of their life cycles (Vrijenhoek 2010; Moalic *et al*. 2012; Rogers *et al*. 2012). The means by which the insular and ultimately ephemeral vent habitats sustain their populations are far from understood, but appear to be governed by a number of physical, historical and biological factors (for review, see Vrijenhoek 2010).

At the geologically isolated back‐arc spreading centre of the East Scotia Ridge, Southern Ocean (ESR; 55°15′S, 29°30′W to 60°30′S, 29°30′W; Fig. S1, Supporting information), the world's coldest ocean is in contact with one of the seafloor's hottest environments. The recent discovery of the E2 and E9 vent fields on the ESR has revealed a new province of vent biogeography, and it has been proposed that environmental and physiological constraints imposed by the Southern Ocean may act as a dispersal filter for vent taxa that possess planktotrophic (feeding) larvae (Rogers *et al*. 2012). The same constraints are thought to account for high endemism and *in situ* evolution of the Antarctic benthos (Brandt *et al*. 2007), among which invertebrates exhibiting high reproductive investment in fewer, larger eggs, with lecithotrophic development, prevail (Thorson 1950; Thatje 2012).

Both vent sites on the ESR are visually dominated by a new species of anomuran, *Kiwa tyleri* (S. Thatje, L. Marsh, C.N. Roterman, M.N. Mavrogordato, & K. Linse under review) at abundances exceeding 700 individuals m^−2^ (Marsh *et al*. 2012). The ‘*Kiwa*’ assemblages around the vents have been divided into three subtypes, based on the average size class of individuals and proximity to fluid exits, with individuals also observed outside the direct influence of hydrothermal fluid in the vent periphery (Marsh *et al*. 2012; Rogers *et al*. 2012). Hydrothermal vents are typically characterized by high abundance of a few species, in contrast to non‐chemosynthetic soft‐sediment deep‐sea benthos. The deep‐sea floor south of the polar front, however, is notable for its scarcity of decapod fauna (Thatje & Arntz 2004). Explanations for a general absence of reptant decapods include their inability to regulate Mg^2+^ haemolymph concentrations below that of seawater, resulting in a loss of activity and paralysis at polar temperatures (Frederich, Sartoris & Pörtner 2001). Despite being in the coldest ocean, the E2 and E9 vent fields are further distinguished by a difference in local hydrographic conditions. The more southern E9 vent field is influenced by lower Weddell Sea Deep Water with ambient water temperature typically at −1·3 °C and therefore colder than its northern counterpart at E2 (*c*. 0·00 °C; Rogers *et al*. 2012).

Other species of *Kiwa* occur in chemosynthetic environments north of the Antarctic Convergence, but in contrasting abundance and distribution to the aggregations at vents on the ESR. *Kiwa hirsuta* occurs at low population densities (0·1–0·2 individuals m^−2^) towards the periphery of the vent fields in the SE Pacific Ocean (MacPherson, Jones & Segonzac 2005). *Kiwa puravida* occurs at cold seeps on the Costa Rica margin, but has also not been observed in extensive aggregations (Thurber, Jones & Schnabel 2011). On the Southwest Indian Ridge, specimens morphologically similar but phylogenetically distinct to the Antarctic *K. tyleri* (Roterman *et al*. 2013) have also been found in close proximity to active vent sources, though at population densities at least an order of magnitude lower than those observed at the Southern Ocean vent fields (Copley 2011). The only other known vent‐endemic anomuran to occur in comparably high‐density populations is the galatheid *Shinkaia crosnieri* from hydrothermal vents in the southern Okinawa Trough. Assemblages of *S. crosnieri,* however, show no evidence of assemblage structure, with juveniles and large mature adults co‐occurring (Tsuchida, Fujiwara & Fujikura 2003).

In this paper, we reconstruct the female reproductive cycle of the Southern Ocean Kiwaidae from both the E2 and E9 vent fields in relation to ecological and physiological constraints in this Antarctic hydrothermal environment. By determining sex ratios, assessing population structure and the reproductive condition of females, we elucidate the controls on defining the ‘*Kiwa*’ assemblages. We hypothesize that the inherent thermal nature of the ESR hydrothermal vents allows this species to exist; however, on leaving the elevated thermal conditions of the vent environment, individuals and their offspring are faced with the physiological constraints imposed by the polar environment, where successful dispersal and survival of larvae are key to maintaining local populations.

## Materials and methods

### Vent Field Descriptions

The vent field at the E2 segment (situated between 56°05·29′ and 56°05·49′S and 30°19·00′ and 30°19·36′W, Fig. S1, Supporting information) is located at *c*. 2600 m depth and consists of active and inactive hydrothermal structures, clustered in a band running approximately north‐west–south‐east along the ESR spreading axis. At the northern limit of the ventfield, ‘Anemone Field’ is situated among basalt pillows on the western most edge of the fissure. Twenty‐five metres due south, the ‘Dog's Head’ vent structure forms the largest (*c*. 12 m) actively venting sulphide edifice comprised of three ‘black‐smoker’ chimneys (maximum recorded vent fluid temperature, 352·6 °C; Rogers *et al*. 2012) with a number of additional high‐temperature ‘black‐smoker’ exits, flanges and beehive structures. To the south of the site, around the periphery of another active high‐temperature chimney, ‘pockets’ of diffuse flow (3·5–19·9 °C; Rogers *et al*. 2012) are found emanating from between basalt pillows.

At a depth of *c*. 2400 m, the E9 vent field is located at the southern end of the ESR between 60°02·50′ and 60°03·00′S and 29°59·00′ and 29°58·60′W (Fig. S1, Supporting information). The distribution of active and inactive vent chimneys within the field appears to be associated with fissures parallel to the ridge axis, running north‐northwest from the edge of the caldera across a seafloor of predominantly flat sheet lavas (Rogers *et al*. 2012). At the northern limit of the vent field, two active chimney structures occur in close proximity. ‘Black & White’ is a *c*. 10‐m‐high structure with multiple ‘black‐smoker’ sources at its summit, emitting fluids with a maximum measured temperature of 380·2 °C (Marsh *et al*. 2012). Lower down the structure, flanges and beehives provide additional exits for hydrothermal fluids at lower temperatures. The southern area of the vent field is characterized by active and extinct chimneys and diffuse flow fields distributed parallel to the ridge axis. The ‘Marsh Towers’ structure consists of two chimneys, rising from a sulphide platform, which emit diffuse flow, but no visible black‐smoker venting. ‘Marshland’, an area of diffuse flow from fissures in basalts, lies immediately to the west.

### Field Sampling

Specimens of *K. tyleri* were collected from six biological sampling dives at the E2 and E9 vent fields during *RRS James Cook* research cruise 42 (7 January–24 February 2010) using the *Isis* remotely operated vehicle (ROV) equipped with a suction sampler. Discrete spatial samples were targeted to investigate the fine‐scale spatial variation in reproductive biology. Three biological sampling dives were conducted at the E2 vent field and a further three sampling dives at the E9 vent field (Fig. S2, Supporting information; Table 1). Spatial samples were kept separate using different chambers of the suction sampler carousel and closeable bioboxes onboard the ROV.

**Table 1:** Sample and population data for *Kiwa tyleri* collected during the 2010 *Isis* ROV dive campaign to the Southern Ocean vent fields

Vent field	*Isis* ROV dive	Vent descriptor	*Kiwa* assemblage sampled	Depth (m)	Latitude (S)	Longitude (W)	Date	*K. tyleri*	Sex			Population statistics	
									M	F	Ratio M : F	χ^2^	Significance
E2	130	Anemone Field	Peripheral	2597	56°05·302	30°19·046	20/01/2010	60	0	60	–	58·01	–
E2	132	Dog's Head	*Kiwa* Assemblage B	2611	56°05·298	30°19·066	22/01/2010	22	8	14	1 : 1·75	1·13	NS
E2	135	Crab City	*Kiwa* assemblage B	2641	56°05·348	30°19·131	25/01/2010	63	25	38	1 : 1·52	2·29	NS
E9	140	Corner of Black and White	*Kiwa* assemblage C	2402	60°02·568	29°58·890	30/01/2010	152	130	22	1 : 0·17	76·74	a
E9	141	Marshland	Base of chimney	2395	60°02·809	29°58·709	30/01/2010	78	35	43	1 : 1·23	0·63	NS
E9	144	Marshland	Peripheral	2398	60°02·822	29°58·722	02/02/2010	33	1	32	1 : 32	28·26	a

M, total males; F, total females; ROV, remotely operated vehicle.

a *P *<* *0·001; NS, not significant.

### Population Structure and Spatial Variations in Sex Ratio

Specimens recovered from the ROV were sorted by sex and ovigerous condition. Sex determination was based on the presence of setose pleopods and the presence of the gonopore on the third pereopod of the females. Females that were carrying eggs were determined as ovigerous, and the brood was carefully removed. The standard measure of body size for anomurans, carapace length (CL), was determined to the nearest 0·01 mm by vernier calipers for both males and females. CL was measured from the midline of the orbital arch to the mid‐dorsal posterior margin of the carapace. Female specimens and eggs for reproductive analysis were then fixed in 4% seawater‐buffered formaldehyde solution before being transferred to 70% ethanol on arrival in the UK.

Further population data were obtained from ROV high‐definition video imagery from the 2010 dive campaign. Using methods outlined in Marsh *et al*. (2013), high‐resolution image mosaics were created of the east and west faces of the ‘Black & White’ chimney (60°02·76′S, 29°58·890′W) at the E9 vent field. ‘*Kiwa assemblages’* were identified, defined and demarcated in adobe photoshop cs5 extended (version 12.0 ×64) (Marsh *et al*. 2012). Using the 0·1‐m laser scale visible in the video imagery, CLs were measured for each ‘*Kiwa assemblage’* type (‘*A*’, ‘*B*’ and ‘*C*’). Measurements were drawn from the raw stills image sequence used to generate the mosaics to avoid artefacts from the mosaicing process.

### Synchronicity of Oocyte Development

Adult females were dissected, and reproductive maturity was assessed by direct visual observation of ovary morphology, colour and relative size under a stereomicroscope. Based on these observations, females were classified into four categories of ovary maturity (Table 2a; based on those described by Dellatorre & Baron 2008, for *Munida gregaria*).

**Table 2:** Subjective stages used to define ovary maturity, reproductive and embryonic development. (a) Morphological variables used to define OMS of female specimens of *Kiwa tyleri*; (b) Morphological variables used to characterize RDS; (c) Stages of EDS

Morphological variable	Ovary Maturity Stage (OMS)			
	OMS 1	OMS 2	OMS 3	OMS 4
(a)				
Position	Two filaments lateral to the hindgut	Begin to proliferate dorsolateral to the hindgut	Dorsolateral to the hindgut	Dorsolateral to the hindgut
Relative size	Detectable under dissecting microscope	In large specimens, detectable to the naked eye	Circular in shape. Conspicuous in the cephalothoracic cavity	Densely packed, irregularly shaped. Occupying most of the cephalothoracic cavity
Pre‐vitellogenic oocytes (po)	Pink	White	–	White
Early vitellogenic oocytes (evo)	Not present	White/yellow	White/yellow	–
Late vitellogenic oocytes (vo)	Not present	Not present	Yellow/orange	Yellow/orange

Morphological variable	Reproductive Development Stage (RDS)			
	RDS 1	RDS 2	RDS 3	RDS 4
(b)				
Moult stage	Soft carapace, white in colour, no evidence of necrosis, no generation of next carapace	Carapace hard, white or yellow in colour, maybe some evidence of early necrosis, no generation of next carapace	Carapace hard, yellow in colour, evidence of necrosis, no generation of next carapace	Carapace, yellow in colour, necrotic carapace evident^a^, generation of next carapace evident
Ovary stage	OM3/OM4 (OM1)	OM4	OM2	OM2/OM3
Ovigerous stage	Not brooding, no egg stalks remaining on pleopods	Not brooding, no egg stalks remaining on pleopods	Brooding (ED1–ED4, see below)	Not brooding, egg stalk remnants on pleopods

	Embryonic Development Stage (EDS)			
	EDS 1	EDS 2	EDS 3	EDS 4
(c)				
Description under examination of light microscope	Translucent spherical eggs with no discernible embryonic development	Opaque spherical eggs with no discernible embryonic development	Eggs ovoid in shape. Embryo occupying approximately half the egg	Embryos ready to hatch or egg mass partially hatched

a At the E9 vent field necrosis is replaced by hydrothermal deposition on carapace causing discoloration.

To obtain oocyte size‐frequency distributions, ovaries were removed by dissection and subsequently imaged using a Leica EZ4HD stereomicroscope (Leica Microsystems, Wetzlar, Germany). It was not possible to separate individual oocytes without rupturing the oocyte structure. Sections of the gonad were therefore laid as flat as possible onto a Petri dish. For inter‐ and intraspecies comparisons, a minimum of 20 Equivalent Circular Diameters (ECDs) of both pre‐vitellogenic and vitellogenic oocytes were then calculated from calibrated images using imageJ (version 1.440 NIMH, Bethesda, Maryland, USA) image analysis software. ECD represents the diameter of a hypothetical circle of equal area to the object measured and therefore takes account irregularities in the shape of the oocytes as a result of dense packing in the ovary. The vitellogenic oocytes were not suitable for histological analysis because of their exceptionally large size (maximum measured oocyte 1558 μm) and high neutral lipid content. Subsamples of ovaries from each spatial sample were, however, examined histologically to confirm pre‐vitellogenic development. A small section of the ovary was dehydrated through a series of graded alcohol solutions and cleared in xylene. The tissues was embedded in paraffin wax and sectioned by microtome at 7 μm. Sections were mounted on glass slides and stained with haematoxylin and eosin.

### Spatial Variation in Reproductive Maturity

During dissection, criteria presented in Table 2b were used to assign a reproductive development stage (RDS) to each female: (i) moult stage: most aspects of life history of crustaceans are to some extent synchronized to the moult cycle (Chang 1995); therefore, to try to begin to understand the life‐history biology of *K. tyleri*, a moult stage has been derived based on the ‘state’ of the external carapace and the presence of next‐generation carapace from dissection; (ii) ovary maturity stage (OMS): assessed by the characteristics described in Table 2a; and (iii) ovigerous condition: in the field, females were classified as either ovigerous (egg carrying) or non‐ovigerous. In the laboratory, a further classification was made whereby the female may no longer be carrying a brood; however, the presence of chitinous remnants (eggs stalks) on the developed pleopods suggests evidence of individual having recently released their brood. These individuals are subsequently termed ‘post‐ovigerous’.

### Embryonic Development and Fecundity

The eggs were staged based on the criteria presented in Table 2c (modified from Pinheiro & Hattori 2003; Dellatorre & Baron 2008). Broadly, embryonic development in the Antarctic Kiwaidae appears to be analogous with development in other anomurans (for review, see Baba *et al*. 2011); however, the most notable feature in late stages of embryonic development of many Decapoda, the pigmented optical lobe (eye), is absent (Thatje *et al*. 2015). Realised fecundity has been defined as the number of eggs carried on the pleopods of each individual (Ramirez‐Llodra 2002). To examine fecundity, the eggs from ovigerous females were enumerated and imaged with the Leica MZ16 (Leica Microsystems, Wetzlar, Germany). Eggs that had not ruptured and had retained the embryonic membrane were measured [egg length (EL) and egg width (EW)] using imageJ (version 1.440). Late‐stage eggs take a prolate ellipsoid shape, and therefore, egg volume (EV) was estimated as EV = 4/3π(EL/2) × (EW/2)^2^ (Baba *et al*. 2011).

## Results

### Population Structure and Spatial Variation in Sex Ratio

**Figure 1:**
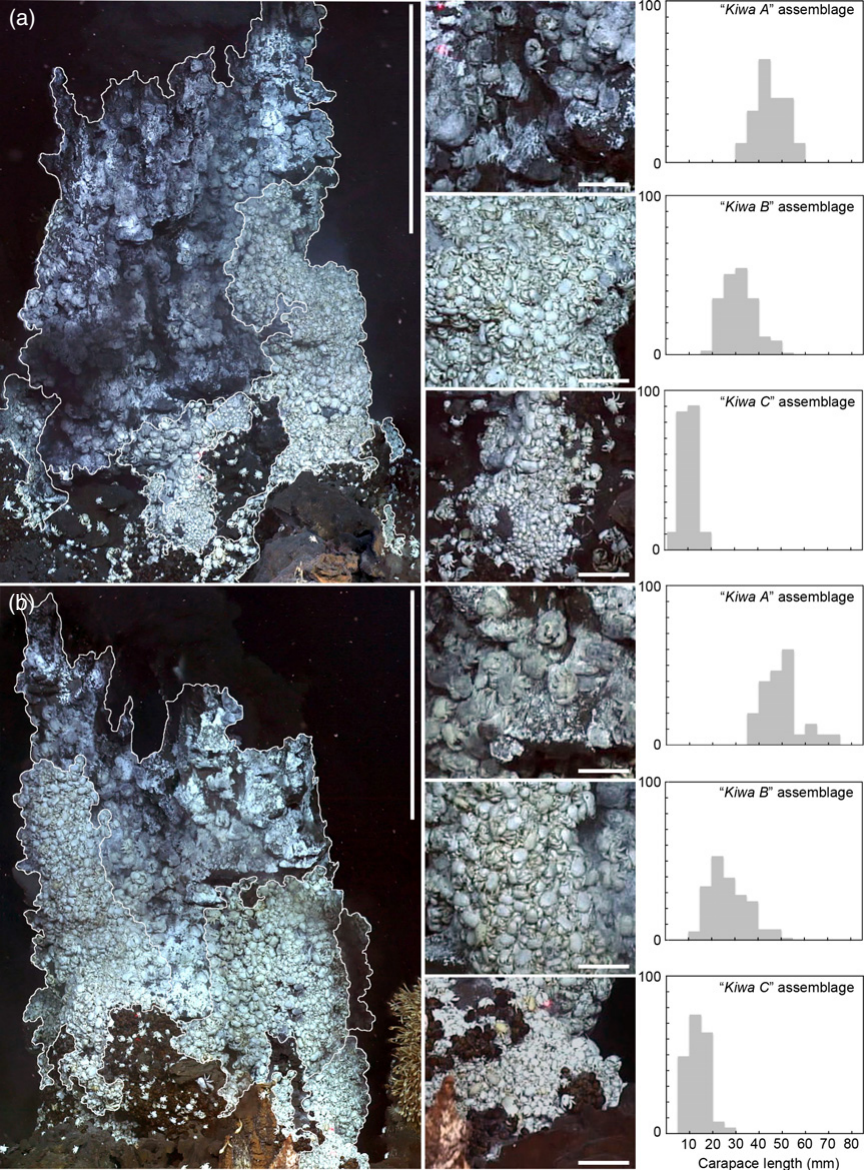
High‐definition image mosaics of the E9 ‘Black & White’ chimney structure. (a) west face remotely operated vehicle (ROV) heading 090; (b) east face ROV heading 270. Percentage size‐frequency distributions are presented for each assemblage type on each face. *Kiwa tyleri* carapace length (CL) was examined digitally from the high‐definition still captures. Chimney scale bar = 1 m; assemblage scale bar = 10 cm.

Using high‐definition video imagery and a parallel‐laser scale, size‐frequency distribution data were obtained from the ‘Black & White’ chimney at the E9 vent field (Fig. 1). The size‐frequency distributions of the three ‘*Kiwa assemblages*’ types varied significantly (Kruskal–Wallis; *H *=* *327·04, *P *<* *0·001; mean CL ± SD ‘*Kiwa A*’ assemblage 47 ± 0·8 mm; ‘*Kiwa B*’ assemblage 30 ± 0·8 mm; ‘*Kiwa C*’ assemblage 12 ± 0·4 mm; data from Marsh *et al*. 2012; nonparametric tests are applied here and for other similar comparisons to be conservative, because data do not meet the requirements for parametric testing).

A total of 408 specimens of *K. tyleri* were collected from the Southern Ocean hydrothermal vent fields in January 2010. To examine whether there was a significant deviation from a 1 : 1 sex ratio within the spatial samples, the χ^2^ test for homogeneity (with Yate's correction for 1 d.f.) was applied. The overall sampled population from the E2 and E9 vent fields did not deviate significantly from the expected 1 : 1 male‐to‐female sex ratio. However, when the data from discrete spatial samples are examined, sex ratio varies consistently among locations in each vent field (Table 1). Large individuals were collected from the ‘*Kiwa A*’ assemblage at ‘Dog's Head’ for taxonomic descriptions and are therefore not included in this current study. An important observation to note is that all specimens recovered were identified as male. Populations sampled from the ‘*Kiwa B*’ assemblages at ‘Dog's Head’ and at ‘Crab City’ did not deviate significantly from unity. The only sample that showed a significant male bias in the population sampled was the ‘*Kiwa C*’ assemblage from the ‘Black & White’ chimney at the E9 vent field (χ^2^ = 76·74 *P *<* *0·001, 1 d.f.). The specimens sampled from around the base of the E9 chimney complex at ‘Marshland’ did not deviate significantly from the expected 1 : 1 ratio. Samples that were not directly associated with hydrothermal fluid flow and collected away from active hydrothermal vent chimneys, however, exhibited a significant female bias (E2 ‘Anemone Field’ no males present; E9 ‘Marshland Periphery’ χ^2^ = 28·26, *P *<* *0·001, 1 d.f.).

**Figure 2:**
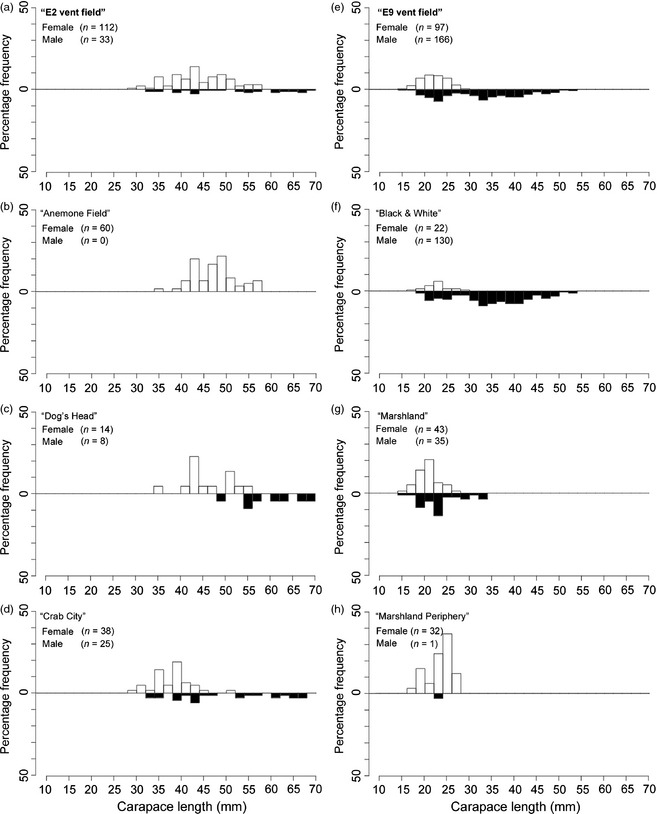
Size‐frequency distributions of *Kiwa tyleri* at the two East Scotia Ridge (ESR) vent fields: E2 left panels and E9 in right panels. White bars (female); black bars (male) (a) E2 vent field; (b) ‘Anemone Field’; (c) ‘*Kiwa B*’ assemblage ‘Dog's Head’; (d) ‘*Kiwa B*’ assemblage ‘Crab City’; (e) E9 vent field; (f) ‘*Kiwa C*’ assemblage at ‘Black & White’; (g) ‘Marshland’; (h) ‘Marshland Periphery’.

Overall, the size of males to females varied significantly for both the E2 and E9 vent fields (Fig. 2; Mann–Whitney rank‐sum test; *T *=* *3106·00, *P *<* *0·001 and *T *=* *7179·50, *P *<* *0·001, respectively) and within each of the spatial samples.

The largest recorded male was from the E2 ‘*Kiwa A*’ assemblage at ‘Dog's Head’ (68·20 mm CL), while the largest recorded female (57·92 mm CL) was sampled from ‘Anemone Field’ at E2. The smallest male and female specimens recovered were both from the E9 vent field, both within the ‘*Kiwa C*’ assemblage at ‘Black &White’ (15·60 and 15·36 mm CL, respectively).

### Spatial Variation in Ovigerous Females

**Figure 3:**
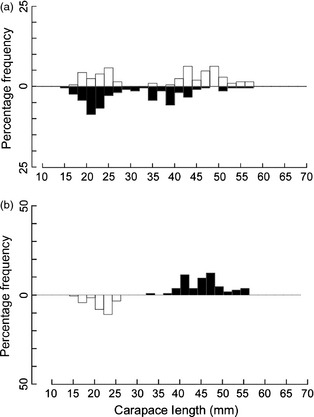
Size‐frequency distributions (a) ovigerous (black bars) and non‐ovigerous (white bars) females from the E2 and E9 vent fields. (b) female *Kiwa tyleri* sampled from the vent periphery at the E2 (‘Anemone Field’; black bars) and E9 (‘Marshland Periphery’; white bars) vent fields.

Females were segregated by their reproductive condition. Spatial variation in the presence of ovigerous females was assessed by comparing the ratio of ovigerous females (brooding or post‐ovigerous) to non‐ovigerous females (Table 3 and Fig. 3a). There is a statistically highly significant association between the proportion of ovigerous to non‐ovigerous females, and location within the vent environment (χ^2^ test for independence = 160·4, *P *<* *0·001, 5 d.f.; Table 3). The smallest ovigerous female (17·6 mm CL) was sampled from ‘Marshland Periphery’ at the E9 vent field and the largest (57·92 mm CL) from E2′s ‘Anemone Field’. Ovigerous females are present throughout the entire size‐frequency distribution.

**Table 3:** Sample and population data for female *Kiwa tyleri* only. Numbers in brackets indicate the number of females dissected for reproductive analysis

Vent field	Vent Descriptor	*Isis* ROV Dive	*Kiwa* n.sp. sampled			TF	Ratio TOF : NOF
			OF	POVF	NOF		
E2	Anemone Field	130	18 (11)	41 (8)	1 (1)	60	59 : 1
E2	Dog's Head	132	1 (1)	0 (0)	13 (10)	14	0·07 : 1
E2	Crab City	135	2 (2)	1 (1)	35 (11)	38	0·09 : 1
E9	Corner of Black and White	140	0 (0)	0 (0)	22 (12)	22	–
E9	Marshland	141	8 (8)	0 (0)	35 (10)	43	0·1 : 1
E9	Marshland (periphery)	144	26 (11)	5 (4)	1 (1)	32	31 : 1

OF, ovigerous females; POVF, females post‐ovigerous; NOF, non‐ovigerous females; TF, total females; TOF, total ovigerous and post‐ovigerous females; ROV, remotely operated vehicle.

Comparable spatial samples were collected from both the E2 and E9 vent fields. The samples for ‘Anemone Field’ and ‘Marshland Periphery’ were obtained from areas of the vent field that were not directly associated with hydrothermal fluid flow. These two samples, although spatially, comprised majority ovigerous or post‐ovigerous females (98·3% and 96·9%, respectively); the size‐frequency distributions are significantly different (Fig. 3b; Mann–Whitney rank‐sum test; *T *=* *561·00, *P *<* *0·001; E2: 47·22 mm median CL, IQR 43·28–49·68 mm; E9: 23·88 mm median CL, IQR 22·49–25·13 mm).

### Ovarian Development

The females of *K. tyleri* have m‐shaped paired ovaries situated in the cephalothorax and proliferate within the hepatopancreas, lateral to the hindgut and the latero‐dorsal artery. The ovaries consist of one or two different size cohorts of developing oocytes enveloped by the germinal epithelium. Longitudinal strands of oogonia proliferate from the germinal epithelium and develop into pre‐vitellogenic oocytes. Vitellogenesis initiates when oocytes measure approximately 250 μm in diameter where the ovary then extends dorsolateral to the gut and proliferates dorsally between the posterior and anterior lobes of the hepatopancreas. At secondary vitellogenesis, oocytes packing can extend through the abdomen to the abdominal segments. The ovaries connect to the gonopores situated on the coxae of the third pair of pleopods. Once extruded and fertilized, the embryos form an egg mass attached to pleopods underneath the abdomen.

**Figure 4:**
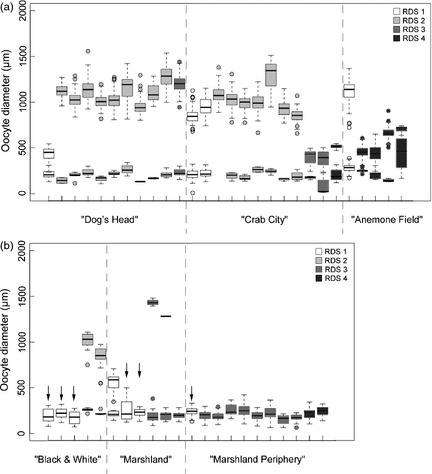
Spatial variations in reproductive development of *Kiwa tyleri*. Oocyte size‐frequency distribution of individual females (denoted by tick marks on the *x*‐axis), for the six spatial samples across the two East Scotia Ridge (ESR) vent fields. (a) E2 vent field (b) E9 vent field. Downward arrow indicates individuals of *K. tyleri* at first oogenesis (OMS 1). OMS, ovary maturity stage.

Of the 408 specimens collected, 209 were female. Ninety‐one of these individuals were then assessed for reproductive analysis (Table 3) and assigned a RDS (Table 2). The minimum requirement of 20 oocytes from each specimen was observed from 50 specimens (Fig. 4). Four OMSs were defined based on the physical appearance of the ovary (Table 2; Fig. S3, Supporting information), and direct measurement of oocytes undertaken in 50 individuals from the six spatial samples across the two vent fields (Fig. 4). The first ovary maturity stage (OMS 1) is defined by the presence of ‘pink’ pre‐vitellogenic oocytes and is further characterized by a unimodal size‐frequency distribution (<300 μm). The minimum reported size at first maturity was 15·36 mm CL from ‘Marshland’ and the E9 vent field. The second ovary maturity stage (OMS 2) is characterized by a bimodal distribution of pre‐vitellogenic (white; *c*. 150–400 μm) and early vitellogenic oocytes (white/yellow; *c*. 400–1300 μm). Two modal components of oocyte diameter are also detected in ovaries at OMS 3 for early vitellogenic oocytes (white/yellow; *c*. 400–1300 μm) and late vitellogenic oocytes (yellow/orange; *c*. 1300–1800 μm). Ovaries at OMS 4 are characterized by a large cohort of late vitellogenic oocytes (yellow/orange; *c*. 1300–1800 μm) and a smaller cohort of pre‐vitellogenic oocytes (white; <300 μm).

### Spatial Variation in Female Reproductive Maturity

Overall, there was a significant difference among the oocyte size‐frequency distributions of samples from different locations within the E2 and E9 vent fields (Figs 4a,b, respectively; Kruskal–Wallis multisample test: E2: *H *=* *274·58, *P *<* *0·001; E9: *H *=* *21·59, *P *<* *0·001). At E2, pairwise multiple comparison procedure (Dunn's method) indicates that the size‐frequency distributions of oocytes were all significantly different (*P *<* *0·05); however, the test statistic *Q* was found to be largest when comparing samples from the spatial extremes from the separate vent fields (E2 ‘Dog's Head’ vs. ‘Anemone Field’ *Q *=* *15·629, *P *<* *0·05). At E9, a significant difference was only observed when comparing the size‐frequency distributions of oocytes from the ‘*Kiwa C*’ assemblage at ‘Black & White’ with ‘Marshland Periphery’ (*Q *=* *4·559, *P *<* *0·05).

From the E2 vent field, specimens of *K. tyleri* that were collected from areas directly influenced by hydrothermal fluid flow showed the highest proportions of females at reproductive development stage 2 (RDS 2) (‘Dog's Head’ 81·8%, *n* = 9; ‘Crab City’ 64·3%, *n* = 9). Specimens at RDS 4 were either not present (‘Dog's Head’, *n* = 0) or in comparatively low proportions (‘Crab City’ 7·1%, *n* = 1). ‘Crab City’ was the only spatial sample to have all four reproductive stages present (RDS 1 14·3%, *n* = 2; RDS 1 4·3%, *n* = 9; RDS 3 14·3%, *n* = 2; RDS 4 7·1%, *n* = 1). The remaining females at ‘Dog's Head’ were at RDS 1 and RDS 3 (9·1%, *n* = 1 and 9·1%, *n* = 1, respectively). The highest proportion of female *K. tyleri* at RDS 3 and RDS 4 were sampled from ‘Anemone Field’ (40·0%, *n* = 8 and 55·0%, *n* = 11, respectively). One individual from this sample had just moulted and was recognized at RDS 1.

At the E9 vent field ‘*Kiwa C*’ assemblage at ‘Black &White’, the highest proportion of females at reproductive development stage 1 (RDS 1) was observed (58·3%, *n* = 7). The remainder of the females within this sample was classified as RDS 2 (41·7%, *n* = 5). At the base of the chimney complex at ‘Marshland’, females in both RDS 1 and RDS 3 stages are present (55·6%, *n* = 10 and 44·4%, *n* = 8, respectively). Only females classified as RDS 3 and RDS 4 were observed at ‘Marshland Peripheral’ (68·8%, *n* = 11 and 31·2%, *n* = 5, respectively).

### Embryonic Development

Four stages of embryonic development were observed across the two vent fields (Table 2; Fig. S4, Supporting information). There is a significant difference in the EV among the four Embryonic Developmental Stages (EDS) (mean ± SD egg volume EDS 1, 1·778 ± 0·234 mm^3^; EDS 2, 1·580 ± 0·267 mm^3^; EDS 3, 2·067 ± 0·312 mm^3^; EDS 4, 2·289 ± 0·330 mm^3^; Kruskal–Wallis; *H *=* *731·91, *P *<* *0·001). There was no significant difference in egg volume, however, between embryos in stage EDS 1 and EDS 2.

### Spatial Variation in Embryonic Development

The highest proportions of ovigerous females were observed from the spatial samples collected from the periphery of the E2 and E9 vent fields (‘Anemone Field’: 30% brooding, 68·3% post‐ovigerous and ‘Marshland Periphery’: 81·3% brooding, 15·6% post‐ovigerous). All of the individuals carrying broods were in the late stages of embryonic development (EDS 4). Within the sample collected around from ‘Marshland’, 18·6% of females were ovigerous, but in various stages of embryonic development (EDS 1, 37·5%; EDS 2, 50%; EDS 4, 12·5%). Samples of *K. tyleri* that were collected from areas directly influenced by hydrothermal fluid flow showed the lowest proportions of ovigerous females all in the early stages of embryonic development (‘Dog's Head’ 7·1%, *n* = 1, EDS 1 and ‘Crab City’ 7·8%, *n* = 2, EDS 3). Specimens collected from the ‘*Kiwa C*’ assemblage at E9 comprised non‐ovigerous females only.

### Fecundity and Embryonic Development

The realised fecundity was determined from 55 ovigerous females sampled from the E2 and E9 vent fields, and the maximum values reported were 175 (41·58 mm CL) and 207 (26·20 mm CL), respectively. Within a brood, the embryos develop synchronously, with all the eggs in one clutch at the same stage. The majority of the eggs examined were at a late stage of development (90% and 76·5% in EDS 4 at E2 and E9, respectively). Clutches from both the E2 and E9 vent fields comprised a high‐proportion of ruptured embryos (42·3% and 37·7%, respectively).

**Figure 5:**
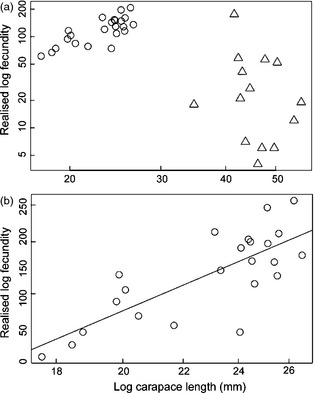
Variation in log fecundity with log carapace length (a) E2 and E9 vent fields. Circles represent E9 and triangles E2; (b) E9 vent field only.

Fecundity was studied in relation to CL independently for the two vent fields (Fig. 5a). Independent of embryonic development stage, there is no significant correlation between CL and fecundity at the E2 vent field (Fig. 5a). At the E9 vent field, however, realized fecundity correlated positively with CL (Fig. 5b; Spearman's rank correlation; ρ = 0·699, *P *=* *0·0002).

Although at the same inferred embryonic development stage (EDS 4), mean volume of embryos varied significantly between the E2 vent field at ‘Anemone Field’ (2·463 ± 0·375 mm^3^) and the E9 vent field at ‘Marshland Periphery’ (2·237 ± 0·297 mm^3^) (Mann–Whitney rank‐sum test; *T *=* *129883·0, *P *<* *0·001).

## Discussion

The ‘black‐smoker’ hydrothermal chimneys at both the E2 and E9 vent fields emit fluids at temperatures exceeding 350 °C (Rogers *et al*. 2012), but a maximum temperature of 24 °C was recorded within the ‘*Kiwa*’ assemblages. Away from sources of active venting, the low temperatures of the deep Southern Ocean prevail (*c*. 0·00 °C and −1·3 °C at E2 and E9, respectively; Rogers *et al*. 2012). Steep thermal and chemical gradients exist at the mixing interface of hot hydrothermal fluid with cold surrounding seawater (Fisher *et al*. 1988; Johnson *et al*. 1988; Shank *et al*. 1998; Sarrazin *et al*. 1999; Luther *et al*. 2001), resulting in high environmental variability over spatial scales of centimetres to metres (Hessler *et al*. 1988; Johnson *et al*. 1988) and temporal scales of seconds to hours (Johnson *et al*. 1988; Copley *et al*. 1999; Cuvelier *et al*. 2011).

The overall chemical composition of the end‐member hydrothermal fluid on the E9 vent field is characterized by higher hydrogen sulphide and lower chloride concentrations compared with fluids measured at active sites at the E2 vent field (James *et al*. 2014). Regardless of the fluid composition, the presence of hydrogen sulphide and oxygen in this seawater/hydrothermal mixing zone enables *in situ* primary production by the epsilon‐ and gamma‐proteobacteria associated with the ventral setae of *K. tyleri* (Reid *et al*. 2013; Zwirglmaier *et al*. 2015). Interestingly, the epsilon‐proteobacteria (the dominant epibiont community associated with E9 Kiwaidae; Zwirglmaier *et al*. 2015) have the ability to use sulphur in both oxidation (electron donors) and respiration (electron acceptors), allowing the additional oxidation of hydrogen for chemolithoautotrophy (Yamamoto & Takai 2011). This may extend the energetically viable habitat for the Kiwaidae available at E9. The gamma‐proteobacteria (the dominant epibiont community associated with E2 Kiwaidae), however, utilize two different sulphur‐oxidizing pathways (Zwirglmaier *et al*. 2015) that may optimize carbon fixation (Yamamoto & Takai 2011) in a energetically narrow zone available at E2. As a result, we suggest regional variations in fluid composition and epibiotic microbial diversity may be key in determining the differences in the extent and proximity of ‘*Kiwa’* assemblages to sources of hydrothermal vent effluent at both the E2 and E9 vent fields.

An ontogenetic change in the diversity of associated microbes has been noted for *K. puravida* at cold seeps (Goffredi *et al*. 2014), An increase in δ^13^C in relation to CL indicates a change in carbon fixation pathways with age and putatively a change in microbial diversity (Zwirglmaier *et al*. 2015). However, this is based on adult stages only and therefore cannot be considered true ontogenetic shift.

In the Southern Ocean, vent habitats may also provide a ‘thermal envelope’ in an otherwise cold‐stenothermal environment from which reptant decapods are otherwise excluded (for discussion, see Thatje & Arntz 2004). As such, there may be strong intraspecific competition for space and nutritional resources among *K. tyleri* within this ‘thermal envelope’ in the Southern Ocean vent environment.

### Spatial Variation in Population Structure and Reproductive Features

**Figure 6:**
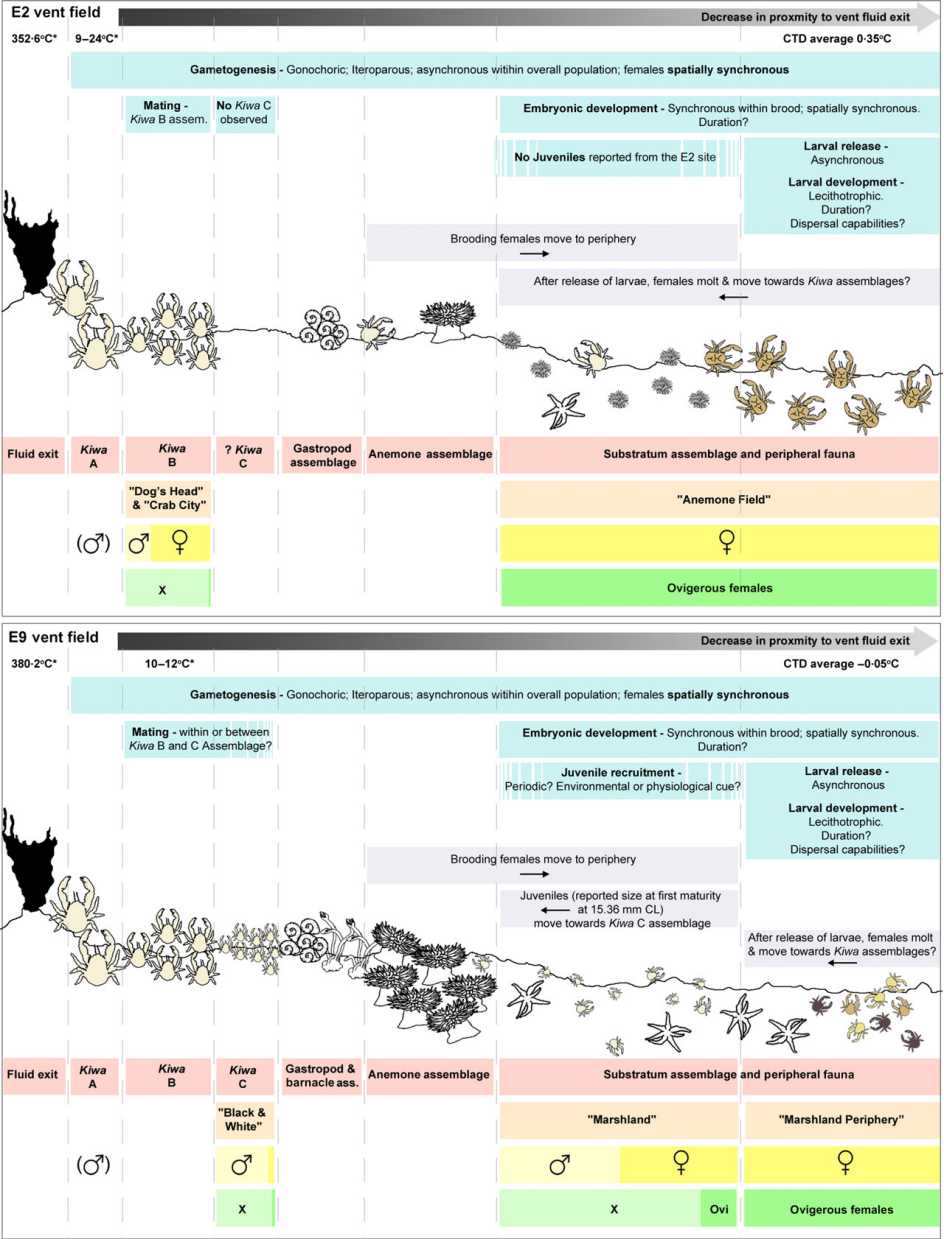
*Kiwa tyleri*. Schematic of the inferred life history based on female reproductive ecology and population structure at the E2 (top) and E9 (bottom) vent fields, Southern Ocean. Temperatures (*) denote remotely operated vehicle (ROV) temperature probe reading. Red bars, assemblage type as defined by Marsh *et al*. (2012); orange bars, spatial sample as defined by this manuscript; yellow bars, sex ratio proportions; in brackets, inferred from taxonomic samples and high‐definition video imagery of chela morphology; green bars, ovigerous condition (*x*; non‐ovigerous); blue bars, reproductive processes (?) indicate unknown parameters; purple bars, movement of *Kiwa tyleri* over reproductive period.

The ‘*Kiwa A*’ assemblage contains the largest bodied (47 ± 0·8 mm) specimens in the vent fields (summarized in Fig. 6). Found in close proximity to high‐temperature fluid exits, this assemblage has the lowest observed population densities (*c*. 65 individuals m^−2^; Marsh *et al*. 2012). From inferred ‘*Kiwa A*’ assemblages on the ‘Dog's Head’ complex at E2, a number of specimens were recovered for taxonomic descriptions. All specimens retrieved were found to be male. Comparing initial chela morphometric data from the specimens recovered from E2 (unpublished data) and high‐definition video imagery acquired from the ‘Black & White’ chimney complex at E9, the ‘*Kiwa A*’ assemblage observed at E9 can also be inferred to be a predominantly male population. Dimorphism in chela size has been observed in shallow‐water squat lobsters (Williams & Brown 1972; Claverie & Smith 2007, 2009) and the vent‐species *S. crosnieri* (Tsuchida, Fujiwara & Fujikura 2003) and is thought to be a consequence of strong sexual selection resulting from male–male competition for receptive females (Tsuchida, Fujiwara & Fujikura 2003; Claverie & Smith 2009; Thiel & Lovrich 2011).

Within the ‘*Kiwa B*’ assemblage, the specimens are significantly smaller than those observed in ‘*Kiwa A*’ (based on HD video footage, 30 ± 0·8 mm CL) and represent higher population densities (*c*. 533 individuals m^−2^; Marsh *et al*. 2012). The majority of females sampled within these assemblages have ripe ovaries in the late stage of vitellogenesis, suggesting that they are receptive or ready to extrude. This is supported by the presence of an ovigerous female in the early stages of embryonic development. Dimorphism in chela size is also apparent in the ‘*Kiwa B*’ assemblage, and ethological studies from high‐definition video footage indicate some antagonistic behaviour within the assemblage, consistent with previous studies (Tsuchida, Fujiwara & Fujikura 2003; Claverie & Smith 2009) suggesting that chela dimorphism in some galatheid squat lobsters could be a result of sexual selection (Video S1, Supporting information).

Still on the chimney, but at a greater distance from fluid exits (>2 m from ‘black‐smoker’ exits; Marsh *et al*. 2012), the highest population densities (*c*. 4017 individuals m^−2^) observed are from the ‘*Kiwa C*’ assemblage and comprise the smallest specimens (12 ± 0·4 mm; Marsh *et al*. 2012). Females examined for reproductive analysis were undergoing the first cycle of gametogenesis or were receptive, with the ovaries in late stages of vitellogenesis.

### Spatial Variation in Females

Many vent‐endemic species exhibit life‐history traits that are phylogenetically conservative (Tyler & Young 1999), and the reproductive traits of *K. tyleri* are broadly similar to those known in other anomurans. The Antarctic *Kiwa* is gonochoric, with the female gonad located in the cephalothorax (S. Thatje, L. Marsh, C.N. Roterman, M.N. Mavrogordato, & K. Linse. under review). The oocyte size‐frequency distributions reveal synchronous gametogenesis and iteroparous reproduction, whereby one cohort of oocytes undergoes vitellogenesis while a second cohort is present, the latter developing only after eggs are extruded onto the pleopods (Perovich *et al*. 2003).

The warmer waters around vent fluid sources at the E2 and E9 vent fields provide an atypical temperature setting for anomurans to maintain populations and reproduce in the Antarctic deep sea. Where the majority of *K. tyleri* reside, thermal conditions are similar to those experienced year‐round by invertebrates in seasonal‐latitude shallow‐water marine environments (Smith *et al*. 2013). However, upon leaving close proximity of the vents, individuals are exposed to the prevailing conditions of the Antarctic, which typically exclude reptant decapods from Southern Ocean waters (Frederich, Sartoris & Pörtner 2001; Thatje & Arntz 2004).

Beyond close proximity of the vent fluid sources, individuals of *K. tyleri* occur at densities at least an order of magnitude lower than those observed in the ‘*Kiwa*’ assemblages. More than half the females sampled here are juveniles, in the initial stages of first gametogenesis; the other half comprised brooding females in the early stages of embryonic development. At E9, this spatial locality therefore appears to be a ‘transition zone’ where juveniles appear to be in search of hydrothermal effluent to sustain their epibiotic bacteria (Reid *et al*. 2013) and brooding females may be actively avoiding the high‐density assemblages and direct influence of hydrothermal fluid flow to protect developing embryos from mechanical damage and/or the hypoxic and sulphidic extremes imposed by the vent environment.

This hypothesis has been proposed for several decapod crustacean species from chemosynthetic environments: the alvinocaridid shrimps *Alvinocaris stactophila* (Copley & Young 2006), *Alvinocaris muricola* (Ramirez‐Llodra & Segonzac 2006) and *Rimicaris exoculata* (Ramirez‐Llodra, Tyler & Copley 2000) and the bythograeid crabs *Bythograea thermydron* (Perovich *et al*. 2003), *Bythograea laubieri* and *Bythograea vrijenhoeki* (Hilário *et al*. 2009). At the E2 and E9 vent fields, females sampled at the greatest distance from high‐temperature hydrothermal influence at ‘Anemone Field’ (E2) and ‘Marshland Periphery’ (E9) further support this hypothesis, with the population comprised predominantly of ovigerous (ovigerous or post‐ovigerous) females in the late stages of embryonic development. Low sample sizes for each female reproductive stage recognized in this study preclude statistical comparisons of the distribution of stages among assemblages, but the pattern shown in the proportions of stages is consistent with this hypothesis, whereby female *K. tyleri* migrate to the periphery of the vent field for brooding.

### Life‐History Traits

At E9, fecundity was positively correlated with CL. Embryos from E2, however, are significantly larger than those sampled from the females at E9 and are reported at a lower fecundity with no correlation with CL. Although both in the late stages of embryonic development, we suggest that the embryos at E2 are more advanced than those from E9 which accounts for the larger size and the observation of ‘partial broods’. At this time, duration of embryonic development is unknown. Species of anomuran crab of the genus *Paralomis*, from the waters of South Georgia, have been shown to have an estimated brooding period of up to 18 months at temperatures of 1·8–3·0 °C (*Paralomis spinosissima*; Reid *et al*. 2007; Thatje & Mestre 2010) and an extended hatching rhythm of larval release over up to 63 days (Reid *et al*. 2007). At discrete spatial localities from which brooding females were sampled at the E2 and E9 vent fields (at lower water temperatures, 0·35 and −0·05 °C, respectively; Fig. 6), we would tentatively suggest that brooding period in *K. tyleri* is >18 months. Populations of females sampled from the periphery show evidence of necrosis (E2) or hydrothermal deposition on the carapace (E9), which also supports the hypothesis of a long brooding period and as a result, a prolonged intermoult period.

The presence of partial broods would indicate that larval release in *K. tyleri* may be asynchronous and prolonged for individuals, as observed in other southern hemisphere lithodid species *Paralomis granulosa* and *Lithodes santolla* that have lecithotrophic larvae (Thatje *et al*. 2003). The environmental conditions faced by individual *K. tyleri* away from vent fluid sources, however, are not constant. Pockets of hydrothermal activity occur in cracked sheet lavas and pillow basalts, and brooding females may experience different environmental conditions. Consequently, although development within broods is synchronous, and spatially synchronous within samples from different locations around the vents, development and larval release may be continuous at the level of the population of the vent field, as a result of this spatial variation in temperature exposure.

Whether the females survive these Antarctic conditions and return to the aggregations around vent fluid exits to mate again, reproducing more than once in a lifetime, remains unclear. The ovigerous females sampled at E2 were significantly larger than those sampled from E9. This could suggest that the females from E2 have completed successive reproductive cycles, consistent with warmer Southern Ocean water (0·5 °C) at E2, which may increase the activity of individuals and their likelihood to return to near‐vent environments following larval release.

### Life Cycle of Males

The life history of male *K. tyleri* at ESR vent fields contrasts with that of the females. Although mating behaviour was not observed directly, the presence of males and receptive females within ‘*Kiwa B*’ and ‘*Kiwa C*’ assemblages suggests that mating may occur among these assemblages.

After mating within the ‘*Kiwa B’* and ‘*Kiwa C*’ assemblages, male specimens of *K. tyleri* would have no requirement to leave the beneficial conditions for nutrition provided by the near‐vent environment, in contrast to brooding females. Continuing to grow in the warmer sulphide‐rich waters of the near‐vent environment, they may attain a larger size than females through successive moults. These large males emigrate or ‘move up’ the chimney towards fluid exits. Physiologically tolerable habitat within the ‘*Kiwa A*’ assemblage, however, may be limited as this assemblage is often in the hottest and most environmentally variable area of vent chimneys, with high‐temperature fluid exits on a high‐proportion of surfaces (Marsh *et al*. 2012; Video S2, Supporting information). We hypothesize that the larger males are more tolerant of vent extremes and represent the ‘upper’ limits of distributions being determined by physical tolerances. Although these larger males that dominate the ‘*Kiwa A*’ assemblage appear more physiologically resilient to greater hydrothermal conditions, ethological observations suggest that individuals are capable of sensing the gradients in flow velocity and avoiding the extreme temperatures associated with primary vent fluids (Video S3, Supporting information).

### Phylogeographic and Evolutionary Implications

Today, the benthic faunal assemblages of the Southern Ocean are the result of an extensive and complex evolutionary history (Gage 2004; Brandt *et al*. 2007; Griffiths 2010; Thatje 2012). High endemism and *in situ* evolution of taxa has been driven by geological, hydrographic and physiological isolation events, and the extrusion and intrusion of species (for review, see Thatje, Hillenbrand & Larter 2005b). Within the Antarctic benthos, taxa exhibit a high incidence of brooding species with lecithotrophic development (Thorson 1950). The few species of lithodid anomuran crabs found in Antarctic waters display a pattern of reproductive investment in fewer, larger eggs, with full lecithotrophy in larval development (Anger *et al*. 2004; Thatje *et al*. 2005a). In this study, we find that *K. tyleri* at Southern Ocean vents exhibits similar features of maternal investment, despite a requirement for the larvae of this vent‐endemic species to colonize isolated vent chimneys to settle and survive. The first larval stage of *K. tyleri* resembles a functional and fully developed megalopa, suggestive of demersal living given the lack of functional swimming appendages, and the presence of large yolk reserves facilitating lecithotrophic development (Thatje *et al*. 2015). Duration of lecithotrophic larval development in this species may exceed a year and potentially include early juvenile stages (Thatje *et al*. 2015). A long‐duration larval phase may support colonization of insular vent environments in a species that exhibits the typical low fecundity of an Antarctic anomuran. This does, however, raise the following question: How is colonization of vents over large geographical distances apart achieved? A recent study showed the Kiwaidae spreading into the Southern Ocean from a Pacific origin via a near complete chain of ridge segments through the deep‐water connection of the Drake Passage, approximately 30 Ma, prior to the geographic and physiological isolation of the Southern Ocean (Roterman *et al*. 2013). The ESR is an intermediate spreading centre that consists of nine ridge segments (Leat *et al*. 2000), and although ultimately transient, it provides a more stable evolutionary setting than fast spreading ridges. Today, we know that the E2 and E9 segments host hydrothermal activity; however, the occurrence of past or extant hydrothermal activity at other segments has not been determined. We suggest that historically, the E2 and E9 vent fields were connected via a number of hydrothermal ‘stepping stones’, however, in more recent times, the number of vent fields has dropped, potentially leading to an isolation of the E2 and E9 populations.

Ecological traits of the Antarctic hydrothermal vent environment and developmental traits of the *K. tyleri* larvae may suggest population retention at the scale of vent fields (E2) or even in extremely isolated cases, individual chimneys (E9). At E2, the incidence of chimneys and venting areas is higher than that at E9. As a result, adults of *K. tyleri* are more dispersed across the E2 vent field. At E9, however, chimneys are not only geographically isolated in locality, but also exposed to greater physiological isolation as the colder waters of the Weddell Sea prevail. Here, we observe high‐density ‘*Kiwa*’ assemblages at individual chimneys and ‘pockets’ of activity inferring that E9 is undertaking habitat contraction. Further genetic investigations at the population level are required to test these hypotheses.

### Conclusion

Living at both thermal extremes to decapod crustaceans leads to complex and disjunct life history for *K. tyleri* at the ESR vent fields (summarized in Fig. 6). Female Kiwaidae at the Southern Ocean exploit the chemical and thermal setting of the hydrothermal environment to survive, sustain and undertake oogenesis; however, to protect the more vulnerable, developing embryos from chemical or mechanical damage, brooding females must move offsite into colder waters, away from the direct influence of hydrothermal fluid. These females are now ‘trapped’ in physiological boundary over very short distances. The lecithotrophic and largely immobile demersal larvae are released, but must ultimately settle within the vent environment if they are to survive. Where adult *K. tyleri* are bound to the vent environment for their adult life, the cold temperatures of the Southern Ocean deep‐sea environment may facilitate the length of duration of all ontogenetic developmental stages, leading to the successful location of vents by the larvae. These larvae, however, are limited in their ability to disperse over long distances, resulting in populations that could be locally isolated.

## Supporting information


**Fig. S1.** Location of the E2 and E9 vent fields in the Southern Ocean.

**Fig. S2.** Sampling locations from the Southern Ocean vent fields. All samples collected with the Isis ROV suction sampler. At the E2 vent field (A) “Anemone Field”; (B) “Kiwa B” assemblage”Dog's Head”; (C) “Kiwa B” assemblage “Crab City”; (D)”Kiwa C” assemblage at “Black & White”; (E) “Marshland”; (F) “Marshland Periphery”. All laser scales visible = 10 cm.

**Fig. S3.** Ovary Maturity Stages (OMS). Images of dissected ovary indicating colouration of oocytes. Scale bars = 1000 μm.Maturity stages as presented in Table 2 are as follows (A) OMS 1; (B) OMS 2; (C) OMS 3; (D) OMS 4. Histological sections of ovary.Hp, hepatopancreas; po, pre‐vitellogenic oocytes; evo, early vitellogenic oocyte; vo, vitellogenic oocyte. Scale bars = 100 μm (E) OMS 1; (F) OMS 2; (G) OMS 3; (H) OMS 4.

**Fig. S4.** Embryonic Development Stages (EDS). Scale bars = 1 mm (A) EDS 1; (B) EDS 2; (C) EDS 3; (D) EDS 3; Scales bars = 1 mm (E) EDS 1; (F) EDS 2; (G) EDS 3; (H) EDS 4 (fully ruptured)

**Video S1.** Antagonistic behaviour of male Kiwaidae.00:01–00:18 Large male covered in filamentous bacteria. 00:18–00:41 Two large Kiwaidae shown ‘fighting’; note large chela. 00:42‐01:11 “Kiwa B” assemblage at the “Carwash” chimney (Marsh et al., 2012) at the E9 vent field (10–12°C).

**Video S2.** Physiologically tolerable habitat.00:01–00:12 “Kiwa A” assemblage at the “Black & White” chimney at the E9 vent field. Vent fluid exit temperature ~380·2°C, which drops to <40°C within the “Kiwa A” assemblage. 00:13–00:40 “Kiwa A” assemblage at the “Dog's Head” chimney at the E2vent field. 00:41–01:05 “Kiwa B” assemblage (left) adjacent to “Kiwa A” assemblage (right) at the “Black & White” chimney at the E9 vent field.

**Video S3.** Avoiding the extremes: Individual male Kiwa tyleri reacting to the gradient in fluid flow velocity andavoiding the hot vent fluid (~352·6°C at the E2 chimney “Dog's Head”)
