# Supplementary figures and images for: In hot and cold water: differential life‐history traits are key to success in contrasting thermal deep‐sea environments
Source: J Anim Ecol. 2015 Mar 2;84(4):898–913. doi: 10.1111/1365-2656.12337 (PMC4964920; doi:10.1111/1365-2656.12337)

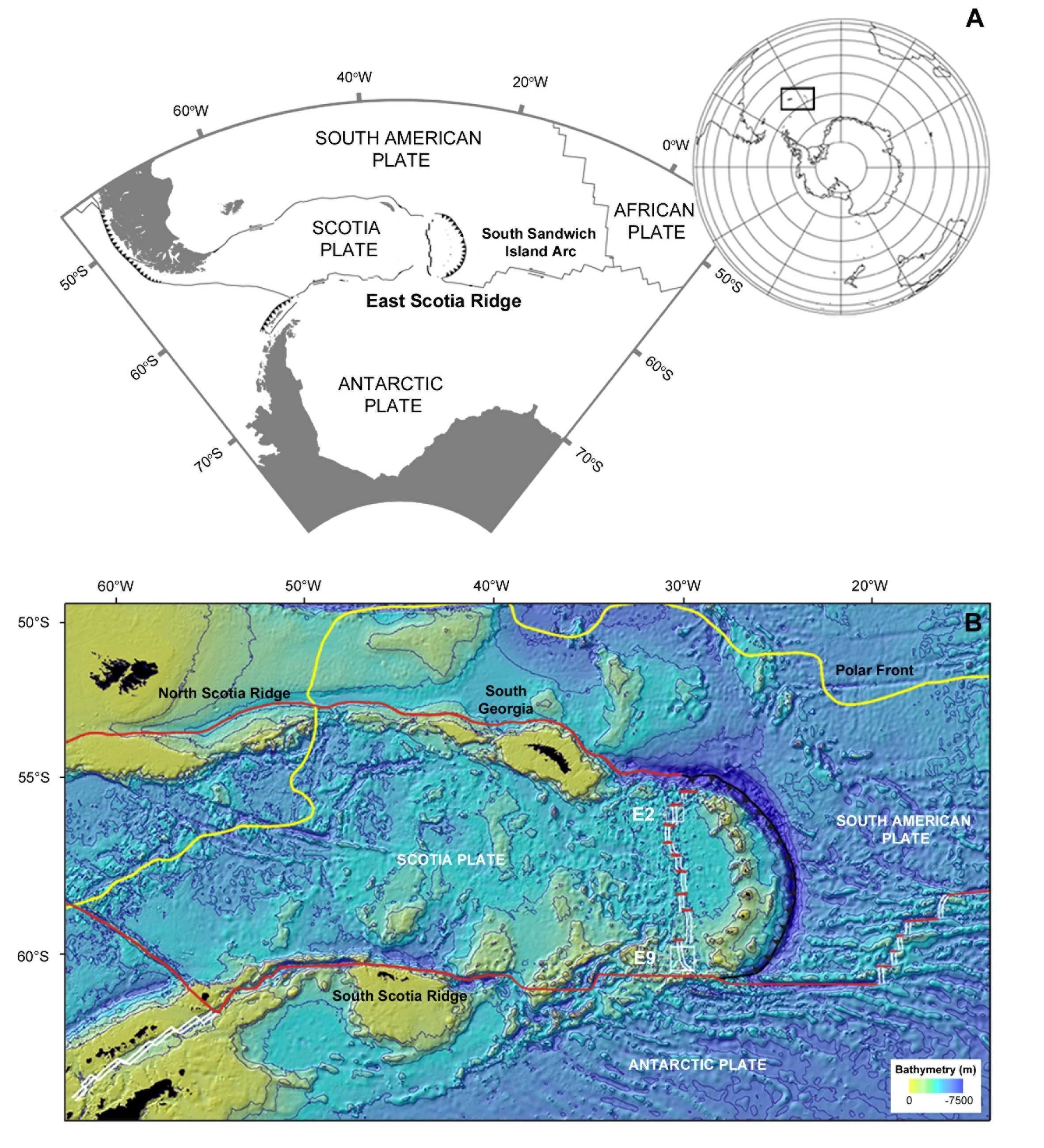

Supplement: Supplementary file 1 — Fig. S1. Location of the E2 and E9 vent fields in the Southern Ocean. [file JANE-84-898-s001.jpg]

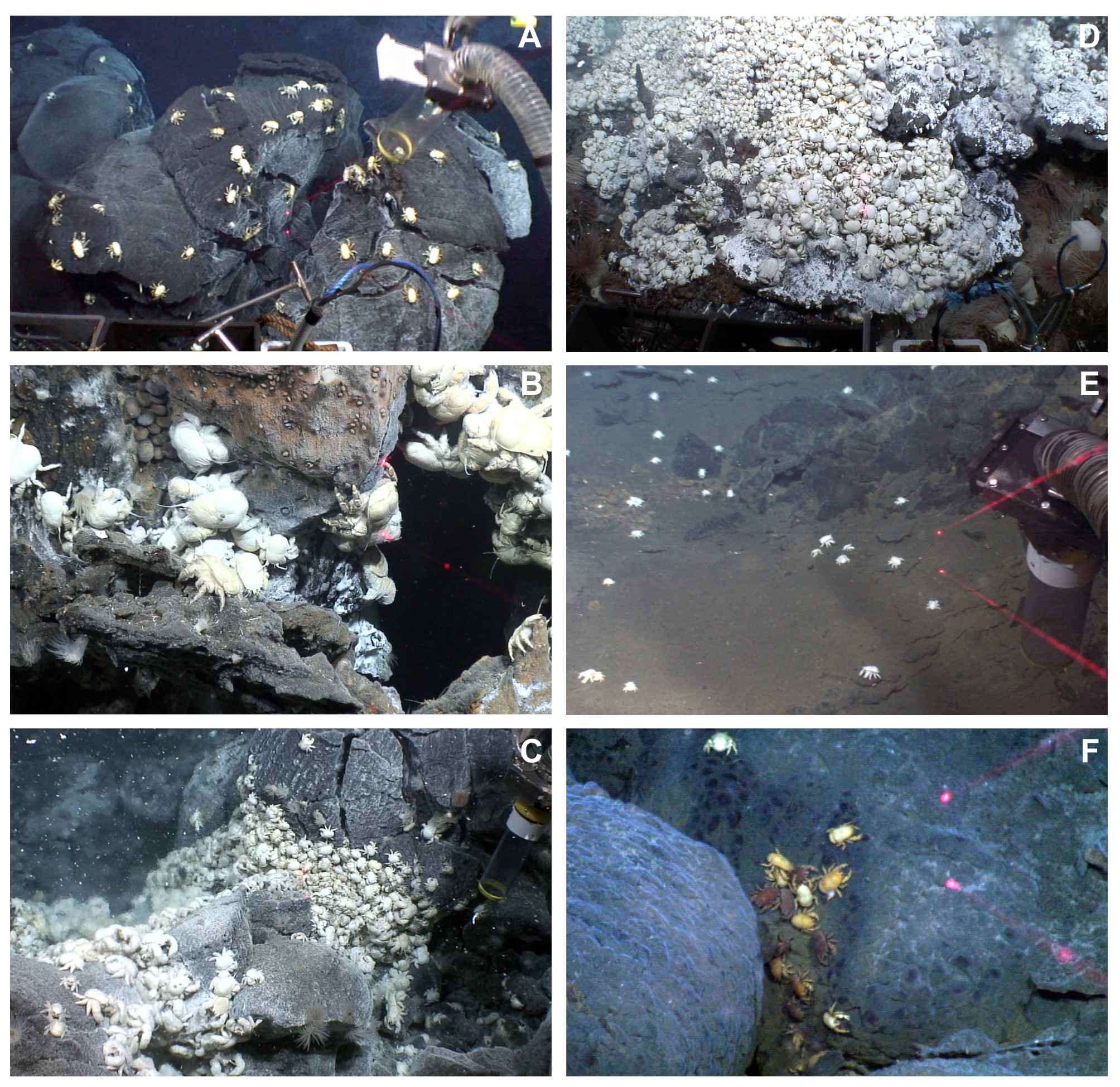

Supplement: Supplementary file 2 — Fig. S2. Sampling locations from the Southern Ocean vent fields. All samples collected with the Isis ROV suction sampler. At the E2 vent field (A) “Anemone Field”; (B) “Kiwa B” assemblage”Dog's Head”; (C) “Kiwa B” assemblage “Crab City”; (D)”Kiwa C” assemblage at “Black & White”; (E) “Marshland”; (F) “Marshland Periphery”. All laser scales visible = 10 cm. [file JANE-84-898-s002.jpg]

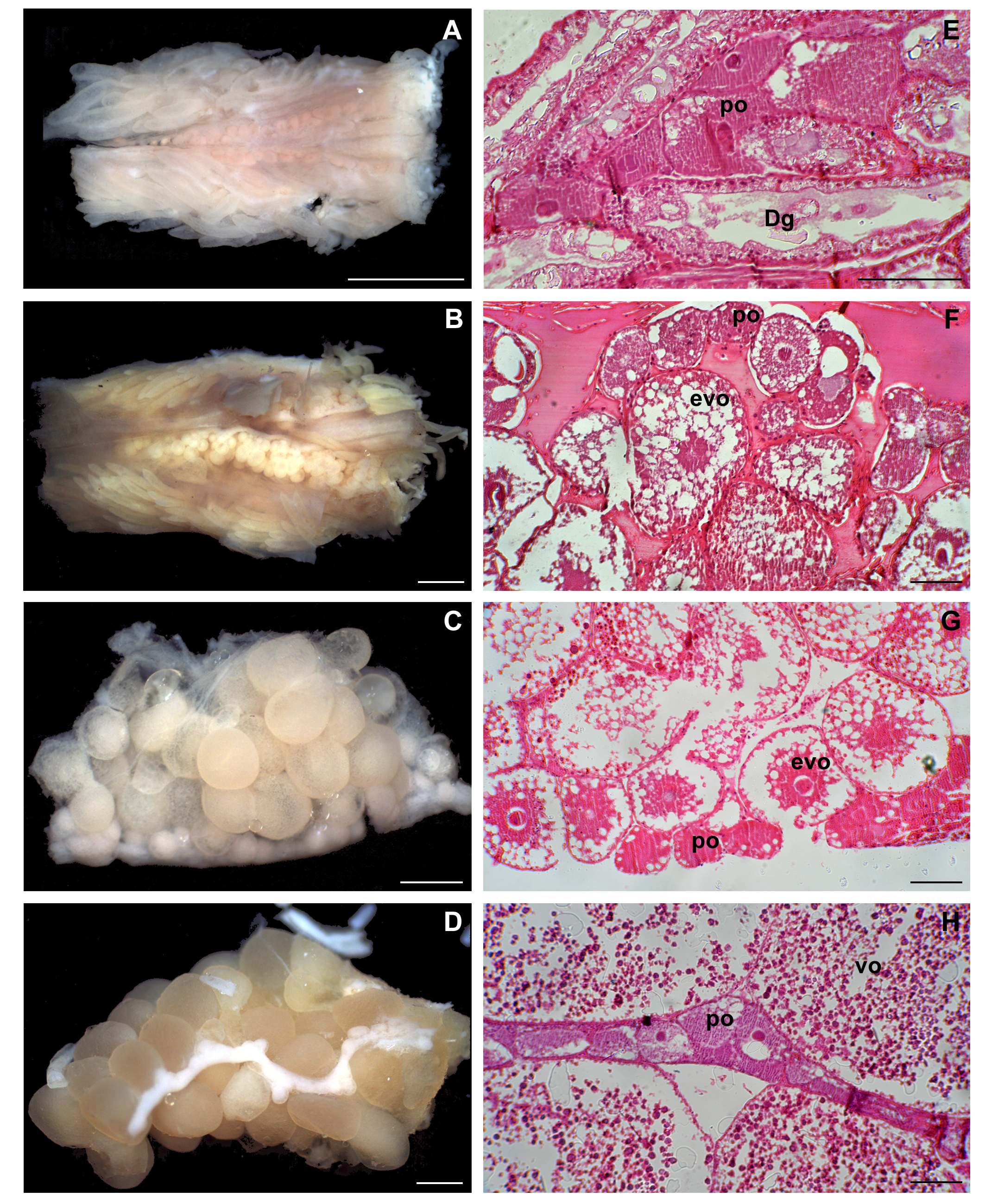

Supplement: Supplementary file 3 — Fig. S3. Ovary Maturity Stages (OMS). Images of dissected ovary indicating colouration of oocytes. Scale bars = 1000 μm.Maturity stages as presented in Table 2 are as follows (A) OMS 1; (B) OMS 2; (C) OMS 3; (D) OMS 4. Histological sections of ovary.Hp, hepatopancreas; po, pre‐vitellogenic oocytes; evo, early vitellogenic oocyte; vo, vitellogenic oocyte. Scale bars = 100 μm (E) OMS 1; (F) OMS 2; (G) OMS 3; (H) OMS 4. [file JANE-84-898-s003.jpg]

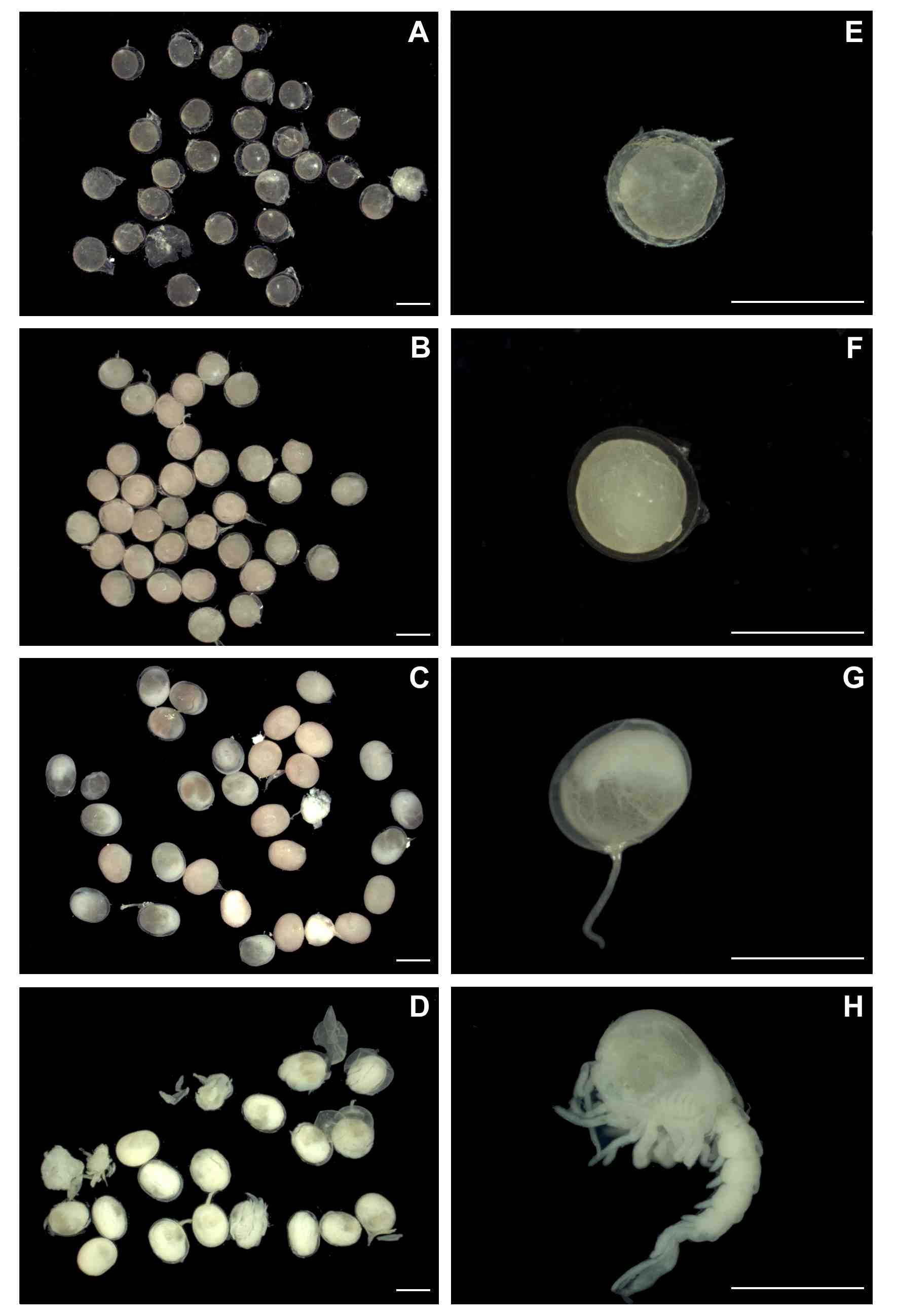

Supplement: Supplementary file 4 — Fig. S4. Embryonic Development Stages (EDS). Scale bars = 1 mm (A) EDS 1; (B) EDS 2; (C) EDS 3; (D) EDS 3; Scales bars = 1 mm (E) EDS 1; (F) EDS 2; (G) EDS 3; (H) EDS 4 (fully ruptured) [file JANE-84-898-s004.jpg]
