# Supplementary material for: In hot and cold water: differential life‐history traits are key to success in contrasting thermal deep‐sea environments
Source: J Anim Ecol. 2015 Mar 2;84(4):898–913. doi: 10.1111/1365-2656.12337 (PMC4964920; doi:10.1111/1365-2656.12337)
Supplement: Supplementary file 8 [file JANE-84-898-s008.docx]

**Supplementary Information**

Figure S1 Location of the E2 and E9 vent fields in the Southern Ocean.

Figure S2 Sampling locations from the Southern Ocean vent fields. All samples collected with the *Isis* ROV suction sampler. At the E2 vent field (A) "Anemone Field"; (B) "*Kiwa B*" assemblage"Dog's Head"; (C) "*Kiwa B*" assemblage "Crab City"; (D)"*Kiwa C*" assemblage at "Black & White"; (E) "Marshland"; (F) "Marshland Periphery". All laser scales visible = 10 cm.

Figure S3 Ovary Maturity Stages (OMS). Images of dissected ovary indicating colouration of oocytes. Scale bars = 1000 μm.Maturity stages as presented in Table 2 are as follows (A) OMS 1; (B) OMS 2; (C) OMS 3; (D) OMS 4. Histological sections of ovary.**Hp**, hepatopancreas; **po**, pre-vitellogenic oocytes; **evo**, early vitellogenic oocyte; **vo**, vitellogenic oocyte. Scale bars = 100 μm (E) OMS 1; (F) OMS 2; (G) OMS 3; (H) OMS 4.

Figure S4 Embryonic Development Stages (EDS). Scale bars = 1 mm (A) EDS 1; (B) EDS 2; (C) EDS 3; (D) EDS 3; Scales bars = 1 mm (E) EDS 1; (F) EDS 2; (G) EDS 3; (H) EDS 4 (fully ruptured)

Video S1 Antagonistic behaviour of male Kiwaidae.00:01-00:18 Large male covered in filamentous bacteria. 00:18-00:41 Two large Kiwaidae shown 'fighting'; note large chela. 00:42-01:11 "*Kiwa B*" assemblage at the "Carwash" chimney (Marsh et al., 2012) at the E9 vent field (10-12°C).

Video S2 Physiologically tolerable habitat.00:01-00:12 "*Kiwa A*" assemblage at the "Black & White" chimney at the E9 vent field. Vent fluid exit temperature ~380.2°C, which drops to <40°C within the "*Kiwa A*" assemblage. 00:13-00:40 "*Kiwa A*" assemblage at the "Dog's Head" chimney at the E2vent field. 00:41-01:05 "*Kiwa B*" assemblage (left) adjacent to "*Kiwa A*" assemblage (right) at the "Black & White" chimney at the E9 vent field.

Video S3 Avoiding the extremes: Individual male *Kiwa tyleri* reacting to the gradient in fluid flow velocity andavoiding the hot vent fluid (~352.6°C at the E2 chimney "Dog's Head")
